# Characterizing the extractable proteins from tomato leaves – A proteomics study

**DOI:** 10.1016/j.fochx.2024.102114

**Published:** 2024-12-21

**Authors:** Marietheres Kleuter, Yafei Yu, Lukas Verdegaal, Francesco Pancaldi, Antoine H.P. America, Atze Jan van der Goot, Luisa M. Trindade

**Affiliations:** a Plant Breeding, Wageningen University, Droevendaalsesteeg 1, 6708, PB, Wageningen, the Netherlands; b Laboratory of Food Process Engineering, Wageningen University, PO Box 17, 6700, AA, Wageningen, the Netherlands; c BU Bioscience, Wageningen Research, Droevendaalsesteeg 1, 6708, PB, Wageningen, the Netherlands

**Keywords:** Protein extraction, Tomato – *Solanum lycopersicum*, Plant development, Agricultural by-products, Proteomics analysis, Soluble proteins

## Abstract

The ambition to utilize agricultural by-products has spotlighted tomato leaves as a promising source for plant-based proteins. High-yielding protein extractability is key for its industrial use, but previous studies reported decreased protein extractability at later stages of plant development. This study investigated the underlying factors in protein extractability through a comprehensive proteomics analysis across four plant developmental stages (vegetative, flowering, fruit-forming, mature-fruit). The findings linked reduced yields to a shift in leaf function, from anabolic to catabolic processes and (a)biotic stress responses. This functional shift is accompanied by decreased protein synthesis and increased protein degradation, leading to an overall decrease of the soluble protein fraction. Furthermore, incomplete extraction of soluble proteins from leaves of later developmental stages, suggested the presence of inhibitory molecules hindering the extraction process. These findings indicate that breeding strategies towards increased amounts of soluble proteins and reduced concentration of inhibitory molecules could enhance protein extraction yields.

## Highlights

• Tomato leaves are a promising source of plant-based proteins.
• Leaf function shifts from anabolic to catabolic processes along development.
• The soluble protein fraction decreases along plant development.
• Endogenous molecules inhibiting protein extraction increase along development.

## 1 Introduction

Meeting the projected future demands of food and proteins requires the identification of novel, sustainable protein sources. A better use of current waste biomass and specific bioresources as novel proteins sources is often suggested as a route to enhance protein supply. Tomato leaves can serve this purpose. With a crude protein content ranging between 19 and 29 % of dry matter (Arab et al., 2019; Liese et al., 2023; Yu, Kleuter, Taghian Dinani, et al., 2023), tomato leaves that normally remain as by-product of tomato cultivation can provide about 3 million ton proteins per year only in the EU (FAOSTAT, 2022; Taylor & Fraser, 2011). However, an efficient protein extraction of all the proteins contained in tomato waste biomass is currently not yet possible (Kleuter, Yu, Pancaldi, Nagtzaam, et al., 2024; Kleuter, Yu, Pancaldi, van der Goot, & Trindade, 2024; Yu, Kleuter, Taghian Dinani, et al., 2023).

To understand the challenges in protein extraction procedures it is important to first understand where proteins are present and what their functions are in the leaves. Several studies pointed out that 80 % of plant proteins are located in the chloroplasts, and these proteins are grouped into soluble and insoluble fractions, with roughly a 50:50 ratio (Fiorentini & Galoppini, 1983). Furthermore, about half of the soluble protein fraction is ribulose-1,5-bisphosphate carboxylase/oxygenase (RuBisCO, EC 4.1.1.39), as the most abundant protein on earth (Barbeau & Kinsella, 1988; Ellis, 1979; Fiorentini & Galoppini, 1983). For tomato, Liese et al. (2023) identified the concentration of RuBisCO being slightly lower than half of the soluble proteins, amounting to about 16 % of the total protein. Following RuBisCO, proteomics analysis in Arabidopsis revealed high abundance of overall photosynthesis associated proteins and photosynthesis antenna proteins in the biomass of dicot plants (Tamary et al., 2019). These two groups of proteins belong predominantly to the insoluble fraction of chloroplast proteins, as they are integrated into the thylakoid membrane. Next to photosynthesis, the chloroplasts also conduct the biosynthesis of several primary and secondary metabolites, including amino acids (Kirk & Leech, 1972), fatty acids (Holzl & Dormann, 2019) and pigments (Joyard et al., 1990; Joyard et al., 2009). Chloroplasts, and with that leaves, conduct such variety of anabolic processes as they serve as source tissues for most of their lifespan by providing energy and metabolites for the rest of the plant (Taiz et al., 2015). At the final developmental stage, known as leaf senescence, degradation of macromolecules, including proteins takes place via various catabolic processes and the resulting nutrients are transferred to sink organs (Guo et al., 2021; Guo & Gan, 2005; Lim et al., 2007). To maintain protein homeostasis, also termed proteostasis, proteins are constantly being synthesized or degraded (Gao et al., 2023). The protein synthesis is performed by a large set of ribosomes, while protein degradation is conducted by several families of protease enzymes and corresponding genes (Gao et al., 2023; Nishimura et al., 2016; Spencer, 1964).

Protein extraction procedures can follow two different aims: 1) extraction of total protein or 2) extraction of specific protein groups. While total protein extraction typically leads to high extraction yields with low purities, extraction focusing on specific protein groups often results in low extraction yields but high purities. Such dilemma between yield and purity is well known for plant proteins extracted from leaves (Tamayo Tenorio et al., 2018). Additionally, the efficiency of protein extractions aiming at recovering the total proteins from tomato leaves has been shown to depend largely on the age of the plant (Kleuter, Yu, Pancaldi, Nagtzaam, et al., 2024; Kleuter, Yu, Pancaldi, van der Goot, & Trindade, 2024; Yu, Kleuter, Taghian Dinani, et al., 2023). With an alkaline protein extraction procedure combined with acid precipitation, the protein extraction decreased from above 0.5 g/g [extracted protein/total protein] for young leaf to below 0.01 g/g [extracted protein/total protein] upon leaf aging. This is partly attributed to changes in the cell walls (Kleuter, Yu, Pancaldi, Nagtzaam, et al., 2024) and partly due to increased protein degradation along development (Kleuter, Yu, Pancaldi, van der Goot, & Trindade, 2024). So far, most of the focus is on protein extraction procedures aiming for pure protein and generally targeting RuBisCO, a plant protein with great functionalities such as gelling (Di Stefano et al., 2018; Nieuwland et al., 2021). When aiming for the extraction of RuBisCO from tomato leaves, extraction purities of up to 76 % have been achieved, however the extraction yield was limited to 0.06–0.10 g/g [extracted protein/total protein] of the total protein content in the leaves. (Liese et al., 2023). Even from the soluble fraction, which is predominantly composed of RuBisCO, only 0.11 to 0.19 g/g [extracted protein/soluble protein] was extracted (Liese et al., 2023). This highlights the complexity of protein extraction from leaves and confirms that achieving high protein purities further reduces the extraction yield.

To understand the factors hindering protein extraction yields better, this study aims to unravel the relationship between protein composition, protein abundances, and functional activities with the final extraction yield in tomato leaves. To do so, a comprehensive proteomics analysis was conducted on four different developmental stages: vegetative growth, flowering, fruit-forming, and mature fruit. By identifying the main processes (GO terms) that determine and thus also change the protein composition throughout the development, new leads for process improvements or breeding targets can be defined to enhance potential utilization of the tomato leaves. In addition, knowing the most abundant proteins per developmental stage allows to develop specific extraction procedures for tomato leaves from different developmental stages.

## 2 Material and methods

### 2.1 Plant material

#### 2.1.1 Harvest

The plant material used for this proteomics study was as described in Kleuter, Yu, Pancaldi, van der Goot, and Trindade (2024). Briefly, seeds of tomato (*Solanum lycopersicum*) cv. Moneymaker from the laboratory of Plant Breeding, Wageningen University, NL, were sown in June 2022 and harvested 119 days after sowing. From every plant, leaves were picked from four developmental stages, representing the vegetative, flowering, fruit-forming, and mature fruit stages. The vegetative stage was defined as the plant section ranging from the top of the plant until the first flower. The flowering stage contained the leaves between the first flower and the first fruit. The fruit-forming stage contained the leaves between the first fruit and the first red-turning fruit (breaker stage). Finally, all the leaves from the first red fruit until the bottom of the plant were depicted as the mature stage. Samples were picked in three biological replicates, representing three distinct individual plants, and directly shock frozen in liquid nitrogen. The frozen leaves were grinded in a coffee grinder while being frozen to allow the use in the proteomics analysis.

### 2.2 Proteomics analysis

#### 2.2.1 Protein and peptide extraction

The extraction procedure aimed at collecting both native proteins and endogenous peptides. Therefore, a single- followed by two- phase extraction method, previously developed by Li et al. (2020), was used for this purpose (with modifications). In short, about 100 mg grinded leaves, 500 μL Methyl-*tert*-butylether (MTBE) and 300 μL of methanol (MeOH) were placed in a reacting tube contained glass beats. Due to the water still present in the grinded leaf tissue, an approximate ratio of 5:3:1 (*v*/v) of MTBE:MeOH:water was generated, giving a single-phase solution after mixing. This mixture was vortexed for 15 min (min) to disrupt the plant tissue and followed by 30 min incubation at room temperature (RT). Next, the material was centrifuged at 16,000*g* for 10 min, leading to the accumulation of soluble components in the supernatant and of insoluble components in the pellet. Subsequently, the supernatant was transferred to a fresh tube. The pellet was washed twice with 500 μL of 90 % MeOH containing 0.1 M tetraethylammonium bromide (TEAB, pH 7) with in-between drying steps conducted in a SpeedVac vacuum concentrator (Thermo Fisher Scientific, Ochten, the Netherlands). The resulting pellet was dissolved in 25 μL 5 % sodium dodecyl sulphate (SDS) with 5 mM Tris(2-carboxyethyl)phosphine (TCEP), 50 mM TEAB (pH 7), and 1 % dithiothreitol (DTT). This step was concluded by 15 min vortexing, 15 min sonication in a 30 °C water bath, and 15 min incubation at 65 °C. Of this fraction, 20 μL were mixed with 2.5 μL iodoacetamide acid (IAA) and digested according to the S-trap protocol (Protifi, Fairport NY, USA). Finally, the Trypsin digested peptides were loaded on the LC-MS/MS.

#### 2.2.2 LC-MS/MS run

The digested proteins were loaded on a liquid chromatography – mass spectrometry (LC-MS/MS) system. The used system was a combination of a M-class ultra-performance liquid chromatography (UPLC) (Waters, Milford, USA) and an QexactivePlus (MS/MS) (ThermoFisher, SanJose, USA). The samples were first loaded on a trap column (NanoTrap RP-2, 10 × 0.075 mm, Phenomenex, Torrance, CA, USA) and subsequently separated over an analytical column (BioZen 2.6 μm Peptide XB-C18 Nano Column, 150 × 0.075 mm, Phenomenex, Torrance, CA, USA). The applied gradient for separation contained 2 % – 35 % acetonitrile in 0.1 % formic acid, running at a flow rate of 350 nL/min for 40 min. This was followed by a washing step, performed by a quick raise to 80 % acetonitrile and back to 2 %, within 15 min. The eluted peptides were sprayed directly into the MS/MS using a NanoFlex electronspray (ESI) source with a spray voltage of 2.4 kV, a capillary temperature of 270 °C, and the S-lens at 60 %. The data was acquired in a data-dependent analysis (DDA) MS/MS selection mode using a top 10 precursor selection method.

#### 2.2.3 Assigning of the peptides

The spectra were processed using FragPipe (version 22.0) (Hsiao et al., 2024) as the overarching workflow, combining the modules MSFragger (version 3.8) (Kong et al., 2017; Teo et al., 2021), Percolator (Kall et al., 2007), ProteinProthet (Nesvizhskii et al., 2003), IonQuant (version 1.9.8) (Yu et al., 2021), and Philosopher (version 5.0) (da Veiga et al., 2020). Specifications for the analysis were match between runs, trypsin cleavage as enzyme parameter, carboxymethyl-cysteine as fixed modification of cysteine and oxidized methionine, as well as acetylated N-terminus and deamidation of glutamine (to pyro-glutamine) as variable modifications. The MS/MS spectra were matched to a subset of the translated *Solanum lycopersicum* genome (ITAG4.0 from Solgenomics network). This step was performed by sub-setting the ITAG4.0 genome to 22,547 protein IDs, as previous transcriptomics analysis revealed such number of expressed genes and it was expected that no proteins would be detected, if also no mRNA was found (Kleuter, Yu, Pancaldi, van der Goot, & Trindade, 2024). The search resulted in matches to 7685 peptides grouped into 1634 distinct protein groups.

#### 2.2.4 Data curation/normalization

For the data analysis, non-imputed Label Free Quantification (LFQ) values received from section 2.2.3 were used. LFQ values allow comparison between samples, as they are normalized for technical variation occurring within the LC-MS/MS system. The raw LFQ data was cleaned for proteins with low counts (< 3), keeping 1404 proteins. This dataset was used for the PCA analysis (2.2.5). Further, outliers were identified using z-score calculation. For each biological replicate, the z-score was calculated by subtracting the mean LFQ value from the individual LFQ value and dividing the result by the standard deviation. Values with z-score higher than 1.1 were considered as outlies and removed from the dataset. Subsequently, proteins with in total less than three LFQ values were removed, leading to a data set containing 1385 proteins. Such dataset was further used for differential abundance analysis (2.2.6, 2.2.7), enrichment analysis (2.2.8), protease identification (2.2.9), and the quantification of the most abundant proteins (2.2.10).

#### 2.2.5 Principal component analysis (PCA)

The PCA was conducted on the LFQ values. Before PCA, the data were logarithmically transformed (log2) to minimize the leverage effect of highly abundant proteins on the PCA outcome. Proteins with missing values were discarded, leading to a dataset containing 721 proteins. The PCA analysis was conducted with the prcomp function in R (version 4.3.0), including the generation of the PCA plot.

Following PCA, the 20 highest and 20 lowest loadings (representing distinct proteins) of the first principal component (PC 1) were used to investigate which proteins (and which biological processes associated to those proteins) drive variability in protein composition across the different developmental stages. The proteinIDs were annotated by the names harbored from the Solgenomics network (ITAG4.0_descriptions.txt) and additional location and functionality were identified by using UniProt (UniProt Consortium, 2023) and protein information at Solgenomics database (https://solgenomics.net/).

#### 2.2.6 Differential abundance analysis over transition stages

To understand which proteins show a significant change in abundance across developmental stages, the log2 fold changes of each protein across consecutive transitions of developmental stages were calculated on the LFQ values (2.2.4). Specifically, this was done for the vegetative to flowering, the flowering to fruit-forming, and the fruit-forming to mature transitions. The log2 fold changes were first defined separately for the biological replicates to minimize plant effects. In a next step, the mean and standard deviation were determined, independently of the number of replicates containing a log2 fold change. Filtering was applied by excluding proteins which had only one log2 fold value across the three comparisons, retaining 1136 proteins. This data was then further used for the Venn diagrams (2.2.7).

#### 2.2.7 Venn diagram

To identify which proteins shared a positive or negative log2 fold change across transitions between subsequent developmental stages, Venn diagrams were generated. For this, the dataset reporting differential abundance with separate biological replicates was used (2.2.6). At first, the distribution of the log2 fold changes per biological replicate were taken to define a threshold of the log2 fold changes representing the top 12.5 % up- and downregulated proteins (Fig. S2, A-C). Such threshold was then applied to subset the differential expressed dataset of the means (Fig. S2, D—F) per transition. These up- and downregulated proteins per transition were then used to generate Venn diagrams. The overlapping proteins between the transitions from vegetative to flowering and flowering to fruit-forming stage were taken further for the enrichment analysis (2.2.8).

#### 2.2.8 Enrichment analysis

**Fig. 1:**
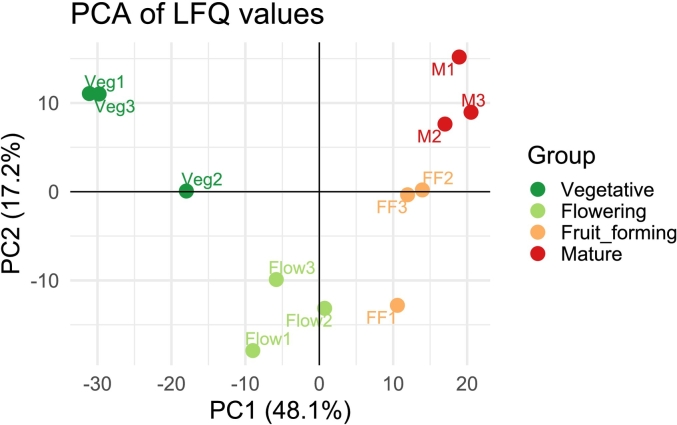
PCA plot for comparison of the different biological replicates. The PCA plot was generated on the LFQ data, clustered by four developmental stages (dark green = Vegetative, light green = Flowering, orange = Fruit-forming, red = Mature fruit) and each replicate is indicated by a number (1,2,3). (For interpretation of the references to colour in this figure legend, the reader is referred to the web version of this article.)

The overlapping proteins between the transitions from vegetative to flowering and flowering to fruit-forming were taken, as the PCA revealed that there are large differences in the PC1 value across these developmental stages, while the fruit-forming, and mature developmental stage shared high similarities (Fig. 1). In the analysis, GO terms had to be present within the datasets of up- and downregulated proteins at least 4 times and PFAM motifs twice. Enrichment was run on GO terms and PFAM motifs, which were annotated on the tomato proteins by respectively using eggNOG 5.0 (Huerta-Cepas et al., 2019) and InterProScan 5 (Jones et al., 2014). Enrichment analysis was conducted with the Fisher's Exact Test in R (version 4.3.0). Additional information on the PFAM motifs were taken from the InterPro platform (Paysan-Lafosse et al., 2023).

#### 2.2.9 Protease identification

Tomato proteases were identified by following the same procedure of (Kleuter, Yu, Pancaldi, van der Goot, & Trindade, 2024). In short, 43 functionally validated Arabidopsis proteases were annotated for their PFAM motifs by using HMM alignments of plant PFAM domains from InterPro database (Blum et al., 2021)and HMMER3 (default parameters) (Mistry et al., 2013), as well as used for a BLAST query against the tomato genome (E = 1E^−3^) (Altschul et al., 1990). The identified tomato BLAST homologs of Arabidopsis proteases were then further filtered based on the PFAM equalities between Arabidopsis queries and tomato subjects. The LFQ dataset was then subsetted for the identified proteases, leading to in total 43 proteases. These 43 proteases were then selected based on their PFAM motifs and the mean of the LFQ values were presented over the different developmental stages.

#### 2.2.10 Quantification of the most abundant proteins

To identify the most abundant proteins within tomato leaves across developmental stages, the LFQ dataset with removed outliers was used (from 2.2.4). At first, the LFQ values were averaged per developmental stage and then the relative abundance was calculated by dividing every mean LFQ value by the mean of the sum of all LFQ values per stage. From such ratios, the 30 most abundant proteins per developmental stage were selected and associated with a functional category (Table S3). The number of proteins associated with an activity are listed in Table S5.

### 2.3 Correlation of soluble proteins and protein extraction yields

To investigate a correlation between the fraction of soluble proteins and the actual protein extraction yields, the soluble fraction was determined as the sum of proteins annotated with the cellular components cytoplasm (GO:0005737) or chloroplast stroma (GO:0009570). The protein extraction yields were taken from the previous study on the same plant material (Kleuter, Yu, Pancaldi, van der Goot, & Trindade, 2024). The correlation between the two parameters was determined by Pearson correlation in R (version 4.3.0). The linear regression and its correlation value were determined by the lm-function, also conducted in R.

## 3 Results

### 3.1 Tomato protein composition varies across developmental stages

To analyze the protein composition of tomato leaves from four different developmental stages (vegetative, flowering, fruit-forming, and mature fruits), a proteomics study was conducted. In the proteomics analysis, 7685 peptides were identified, which were assigned to 1634 distinct proteins. After removal of low count proteins (< 3), a dataset with 1404 proteins remained. To assess the general similarities and differences between the proteins from the different developmental stages, a Principal Component Analysis (PCA) was performed on the LFQ data (Fig. 1). The PCA reduced the dimensionality of the data (i.e., the LFQ values of 721 proteins (after NA removal)), to 3 principal components (PCs) (Fig. S1). The first two PCs, PC1 and PC2, explained 48.1 and 17.2 % of the variation in protein abundance across the leaf samples of the four developmental stages, respectively. Fig. 1 shows the PCA plot of PC1 and PC2, revealing a clear clustering of replicated samples based on developmental stages, highlighting both the accuracy of replicates and the marked differentiation of protein composition of tomato leaves across developmental stages. Specifically, while samples from vegetative and flowering stages are well separated from each other and the other samples along PC1, the fruit-forming samples tend to cluster with the ones from the mature stage, suggesting similar protein composition between the latter two stages with regard to the proteins driving the PC1 patterning.

To understand which protein functions drive the observed differentiation between developmental stages along PC1, the proteins associated to the highest 20 positive and negative loadings of PC1 were extracted (Table S1). Among the proteins showing the strongest negative PC1 loadings, which primarily contribute to the positioning of the vegetative stage samples, 16 out of 20 were proteins perform a function within chloroplasts. Furthermore, this group contained eight ribosomal and four heat shock proteins, three of which were associated with RuBisCO (Solyc01g028810.3, Solyc03g120850.4, Solyc11g069790.2). Most of these proteins can be associated with anabolic processes, such as protein or amino acid synthesis, as well as photosynthesis (marked in green in Table S1). Contrary, among the 20 proteins with the highest positive PC1 loadings, and thus driving the positioning of the samples from fruit-forming and mature stages, only four proteins were located in the chloroplast, while others were located in the cytosol, the extracellular space, or the plasma membrane. Out of these 20 proteins, four were proteases, which drive the catabolic process of protein degradation (marked in blue in Table S1). The other 16 of the 20 proteins displayed a variety of distinct functions, often involved in plant defence or stress responses.

#### 3.1.1 Detailed trends in abundance of different proteins throughout developmental stages

**Fig. 2:**
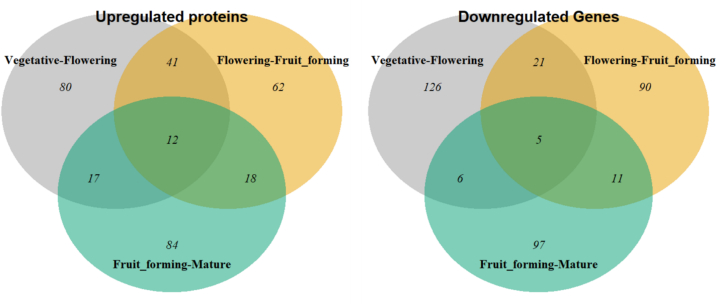
Venn diagrams of the significantly up- and downregulated proteinIDs of the three transitions occurring along four developmental stages namely: Vegetative to Flowering in grey, Flowering to Fruit-forming in orange and Fruit-forming to Mature fruit in cyan. The values in the circles/overlaps indicate the number of proteins.

To understand the differences in the activities of leaves from different developmental stages, a differential abundance analysis of the leaf proteins was performed. This was conducted on the LFQ data after removal of outliers, thus containing 1385 proteins. The analysis compared abundance of the different proteins between consecutive developmental stages. From the vegetative to the flowering stage, 150 proteins were up- and 159 proteins were downregulated. From the flowering to the fruit-forming stage, 133 and 127 and from the fruit-forming to the mature stage 131 and 119 proteins were identified to be up- and downregulated, respectively. To determine proteins that share up- or downregulation across multiple transitions, a Venn-diagram was produced (Fig. 2), showing that 12 proteins were constantly upregulated between subsequent developmental stages from the vegetative to the mature stage, while five were constantly downregulated.

#### 3.1.2 Biological characterization of the changes in protein abundance: Gene ontology and PFAM enrichment analyses

To understand which biological processes are affected by the previously identified up- and downregulated differential abundant proteins, enrichment analysis for gene ontology (GO) and PFAM motifs were performed. The transition between fruit-forming and mature stage was excluded from these analyses because of the limited overall protein differences between these stages as identified in the initial PCA (Fig. 1). The analysis was therefore conducted on the 53 up- and the 26 down-regulated proteins overlapping between the transitions from vegetative to flowering (Vegetative-Flowering) and flowering to fruit-forming (Flowering-Fruit_forming) (Table S2). The results showed 49 significantly enriched GO terms among the upregulated proteins (Table 1) and four enriched GO terms among the downregulated proteins. GO terms can be categorized into cellular component, biological processes, and molecular functions. The enriched GO terms from the upregulated proteins categorized into cellular components were for example chloroplast, including the chloroplast envelope and the plastid and secretory vesicles. Enriched GO terms categorized into molecular functions were mRNA binding, chitinase activity, serine-type-endopeptidase activity, and overall molecular function. The largest category, biological processes, revealed 35 enriched GO terms, such as responses to hormones or stresses, defence against biotic factors or proteolysis. Summarizing the GO terms enriched among the upregulated proteins across development predominantly fell into categories related to various (a)biotic stresses, as well as catabolic processes. The four enriched GO terms on the downregulated proteins identified were nucleolus (GO:0005730) and cytosolic large ribosomal subunit (GO:0022625) as cellular components, RNA binding (GO:0003723) as molecular function and chlorophyll biosynthetic process (GO:0042742) as biological process.

**Table 1:** 49 significantly enriched GO terms identified within the 53 upregulated proteins overlapping between the transition from vegetative to flowering and flowering to fruit-forming.

**GO**	***p*-value**	**GO category**	**GO term short name**
GO:0005576	0.000	cellular_component	extracellular region
GO:0005975	0.000	biological_process	carbohydrate metabolic process
GO:0099503	0.000	cellular_component	secretory vesicle
GO:0004568	0.000	molecular_function	chitinase activity
GO:0033993	0.000	biological_process	response to lipid
GO:0014070	0.000	biological_process	response to organic cyclic compound
GO:0009651	0.000	biological_process	response to salt stress
GO:0006032	0.000	biological_process	chitin catabolic process
GO:0016998	0.000	biological_process	cell wall macromolecule catabolic process
GO:0009617	0.000	biological_process	response to bacterium
GO:0010035	0.000	biological_process	response to inorganic substance
GO:0010262	0.000	biological_process	somatic embryogenesis
GO:0009505	0.000	cellular_component	plant-type cell wall
GO:0009536	0.000	cellular_component	plastid
GO:0006508	0.001	biological_process	proteolysis
GO:0002215	0.001	biological_process	defence response to nematode
GO:0009751	0.001	biological_process	response to salicylic acid
GO:1901701	0.001	biological_process	cellular response to oxygen-containing compound
GO:0005737	0.001	cellular_component	cytoplasm
GO:0008150	0.001	biological_process	biological_process
GO:0009611	0.001	biological_process	response to wounding
GO:0010015	0.002	biological_process	root morphogenesis
GO:0009941	0.002	cellular_component	chloroplast envelope
GO:0050832	0.002	biological_process	defence response to fungus
GO:0006952	0.002	biological_process	defence response
GO:0009627	0.002	biological_process	systemic acquired resistance
GO:0044550	0.003	biological_process	secondary metabolite biosynthetic process
GO:0005615	0.003	cellular_component	extracellular space
GO:0003729	0.003	molecular_function	mRNA binding
GO:0098542	0.003	biological_process	defence response to other organism
GO:0009414	0.004	biological_process	response to water deprivation
GO:0009888	0.004	biological_process	tissue development
GO:0009733	0.006	biological_process	response to auxin
GO:0009753	0.006	biological_process	response to jasmonic acid
GO:0005794	0.006	cellular_component	Golgi apparatus
GO:0031347	0.007	biological_process	regulation of defence response
GO:0071310	0.008	biological_process	cellular response to organic substance
GO:0007165	0.009	biological_process	signal transduction
GO:0003674	0.009	molecular_function	molecular_function
GO:0042742	0.010	biological_process	defence response to bacterium
GO:0009507	0.010	cellular_component	chloroplast
GO:0004252	0.010	molecular_function	serine-type endopeptidase activity
GO:0009809	0.012	biological_process	lignin biosynthetic process
GO:0009744	0.017	biological_process	response to sucrose
GO:0044248	0.021	biological_process	cellular catabolic process
GO:0071456	0.029	biological_process	cellular response to hypoxia
GO:0048589	0.032	biological_process	developmental growth
GO:0005829	0.035	cellular_component	cytosol
GO:0009723	0.037	biological_process	response to ethylene

In addition to the GO enrichment analysis, the same type of analysis was run for PFAM motifs, delivering further insights into the functions of the same 53 upregulated and 26 downregulated proteins. In total, 18 PFAM motifs displayed enrichment among the upregulated proteins (Table 2) and one among the downregulated proteins. Among the 18 PFAM motifs from the upregulated proteins, seven were associated with protein degradation and five with the hydrolysis of carbohydrates. This underlines the results from the GO enrichment that catabolic processes increase along plant development. The only downregulated PFAM was PF00118, representing the TCP-1/cpn60 chaperonin family, associated to prevention of protein misfolding under stress.

**Table 2:** 18 significant PFAM motifs identified within the 53 upregulated proteins overlapping between the transition from vegetative to flowering and flowering to fruit-forming.

**PFAM**	**p-value**	**PFAM name**	**PFAM short description**
PF00182	0.000	Glyco_hydro_19	Chitinase class I
PF00082	0.001	Peptidase_S8	Subtilase family
PF05922	0.001	Inhibitor_I9	Peptidase inhibitor I9
PF17766	0.001	fn3_6	Fibronectin type-III domain
PF00251	0.011	Glyco_hydro_32N	Glycosyl hydrolases family 32 N-terminal domain
PF00704	0.011	Glyco_hydro_18	Glycosyl hydrolases family 18
PF08244	0.011	Glyco_hydro_32C	Glycosyl hydrolases family 32C terminal
PF00188	0.018	CAP	Cysteine-rich secretory protein family
PF00332	0.018	Glyco_hydro_17	Glycosyl hydrolases family 17
PF00141	0.021	peroxidase	Peroxidase
PF00187	0.026	Chitin_bind_1	Chitin recognition protein
PF03051	0.026	Peptidase_C1_2	Peptidase C1-like family
PF00036	0.035	EF-hand_1	EF hand
PF02225	0.035	PA	Protease domain
PF08246	0.035	Inhibitor_I29	Cathepsin propeptide inhibitor domain (I29)
PF13499	0.035	EF-hand_7	EF-hand domain pair
PF00112	0.046	Peptidase_C1	Papain family cysteine protease
PF00560	0.046	LRR_1	Leucine Rich Repeat

#### 3.1.3 Protein abundance of a subset of proteases increased along developmental stages

**Fig. 3:**
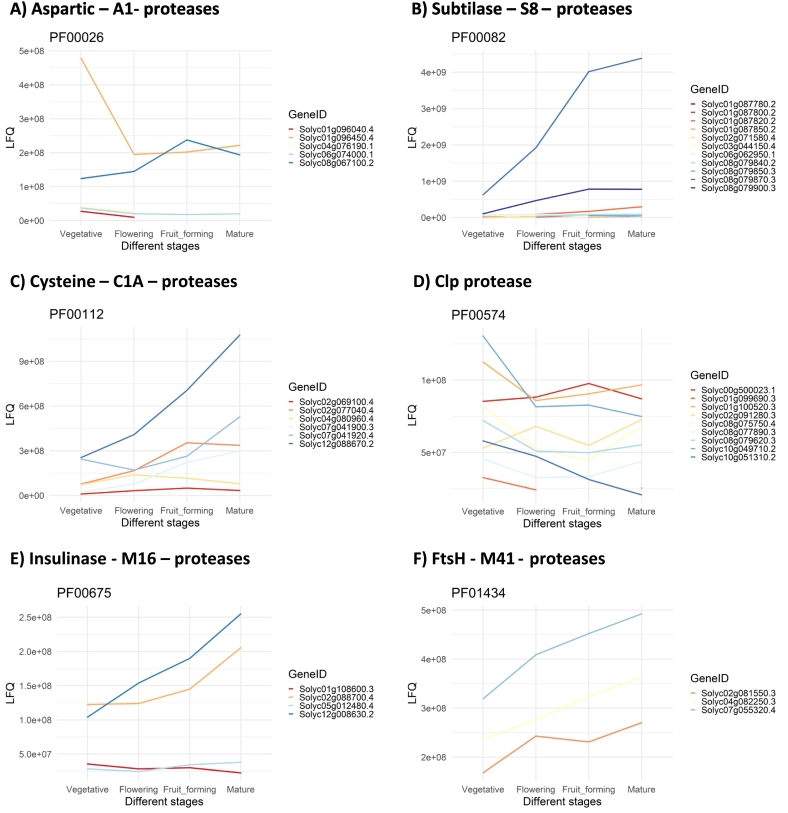
LFQ values of various proteases separated by their protease families: A) PF00026 = aspartic – A1, B) PF00082 = subtilase – S8, C) PF00112 = cysteine – C1, D) PF00574 = Clp, E) PF00675 = Insulinase – M16, and F) PF01434 = FtsH – M41 protease families. The x-axis indicates the different developmental stage (Vegetative, Flowering, Fruit-forming, and Mature) and each line represents a distinct protease. The data shows the mean of the LFQ values.

As proteolysis and distinct protease families were identified in the GO and PFAM enrichment analysis on the upregulated proteins, respectively, a more detailed investigation for proteases was conducted. By doing so, specific proteases can be identified as major drivers of protein degradation along development. Protease genes were identified by a BLAST and PFAM-based search, yielding a set of 43 tomato proteases in the LFQ dataset. The abundance patterns across developmental stages of the protease families with at least three members in the LFQ dataset are displayed in Fig. 3. Protease families showing an increase in abundances across developmental stages included subtilases (Fig. 3 B), cysteine proteases (Fig. 3 C), insulinases (Fig. 3, E), and FtsH proteases (Fig. 3 F). By contrast, the members of the aspartic (Fig. 3 A) and Clp (Fig. 3 D) proteases exhibits predominantly a decrease in their abundances from vegetative to mature stages.

### 3.2 A small number of proteins (<30) represent more than half of the proteins present in tomato leaves

**Fig. 4:**
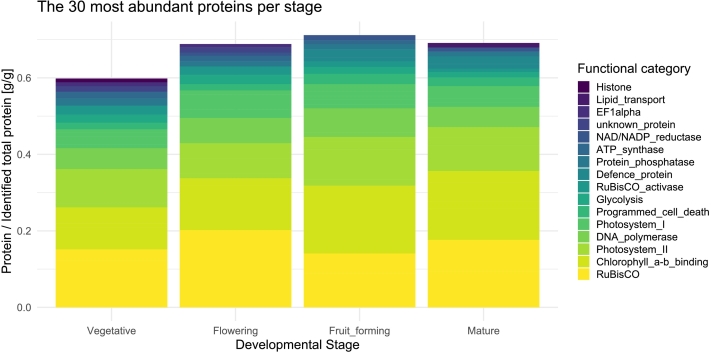
The abundance of the top 30 proteins shown as protein/identified total protein [g/g] per developmental stage (Vegetative, Flowering, Fruit_forming, Mature). Proteins were associated to 16 distinct groups, based on their activity (Table S5).

A further analysis entailed the characterization of the most abundant proteins across the four tomato developmental stages. To this aim, the relative abundances of single proteins at each developmental stage were calculated based on LFQ values (Fig. 4). Overall, the 30 most abundant proteins at each developmental stage made up 0.598, 0.688 0.712, and 0.691 g/g [protein/total identified proteins] at the vegetative, flowering, fruit-forming, and mature fruit stage, respectively. Across all stages, RuBisCO was the most abundant protein, representing 0.152, 0.202, 0.141, and 0.176 g/g [protein/total identified proteins] at each respective stage (Table S4). The total abundance of RuBisCO is represented by two separate proteins: the large subunit (Solyc00g500063.1) and the small subunit (Solyc03g034220.3) (Table S5). Other highly abundant proteins were represented by different photosynthesis-related proteins, including chlorophyll *a*-b binding, photosystem I and photosystem II proteins (Table S5). While the amount of chlorophyll *a*-b binding proteins increased constantly from 0.11 to 0.18 g/g [protein/total identified proteins] across the different stages, the photosystem proteins displayed a wavy pattern in the range of 0.049 to 0.072 g/g (photosystem I) and 0.092 to 0.127 g/g (photosystem II) (Table S4). Further, proteins associated with functions as DNA polymerase, programmed cell death, glycolysis, RuBisCO activase, and more were also overall abundant across developmental stages. In summary, the results just presented highlight that the most highly abundant proteins (in mass terms) present in tomato leaves were, independently of the developmental stage, associated with energy generation. Such energy generation is predominantly carried out within chloroplasts (Table S4). Accordingly, 0.730, 0.760, 0.745, and 0.750 g/g [protein/total identified proteins] proteins from the vegetative, flowering, fruit-forming and mature stage, respectively, were encoded by genes associated to the GO “chloroplast” cellular component (GO:0009507).

### 3.3 The soluble protein fraction decreases along developmental stages and correlates with the protein extraction yield

The final analysis was done to investigate the correlation between soluble proteins and actual protein extraction yields. To achieve this aim, the soluble proteins were estimated by summing up the abundances of proteins annotated with the cellular components cytoplasm (GO:0005737) or chloroplast stroma (GO:0009570). In parallel, the protein extraction yields were taken from the previous study (Kleuter, Yu, Pancaldi, van der Goot, & Trindade, 2024). The concentrations of proteins predicted to be located in the cytoplasm or chloroplast stroma were found to be 0.212 and 0.392, 0.171 and 0.396, 0.158 and 0.318, and 0.142 and 0.349 g/g [proteins/total identified proteins] for the vegetative, flowering, fruit-forming, and mature stages, respectively. Thus, these proteins account for a fraction of soluble protein of 0.605, 0.567, 0.476, and 0.490 g/g [proteins/total identified proteins] for the respective developmental stages. The protein extraction yields decreased along developmental stages from 0.51 to 0.01 g/g [extracted protein/ total protein]. A Pearson correlation analysis between the protein extraction yield and the soluble proteins revealed a moderate positive correlation, with an R^2^ value of 0.85. The actual plot can be found in the supplements (Fig. S4). This result suggests that higher concentrations of soluble proteins in tomato leaves were associated with higher protein extraction yields.

## 4 Discussion

This study aimed at dissecting the relationship between composition, abundance, and functional activity of the proteins found in tomato leaves and the protein extraction yields. To meet this objective, a comprehensive proteomics analysis was conducted on the proteins from tomato leaves of four different developmental stages: vegetative growth, flowering, fruit-forming, and mature fruit. In recent studies, it was shown that the protein extraction yield decreases along developmental stages, which was partly explained by changes in the plant cell walls (Kleuter, Yu, Pancaldi, Nagtzaam, et al., 2024) and by an increase in the activity of proteases (Kleuter, Yu, Pancaldi, van der Goot, & Trindade, 2024) throughout plant development. In this perspective, understanding which biological processes, from a proteomic point of view, take place along tomato development can help to identify further potential drivers for such decline. In addition, characterizing the changes in abundance of specific proteins across developmental stages can give insights into possible optimization steps in extraction protocols. The results of our proteomic study revealed that proteins within tomato leaves change in their activity from anabolic processes in the early developmental stages to catabolic and defence processes in later developmental stages. Moreover, the fraction of soluble proteins decreases along plant development, thus correlating with the decrease in protein extraction yield. Finally, it was found that less than 30 proteins account for more than half of the total protein mass. Of these 30 proteins, 22 are located in the chloroplasts and participate in photosynthesis or energy generation. In this section, the findings related to enhancing protein extraction yields from tomato leaves are discussed.

### 4.1 The activity of tomato leaves changes from anabolic processes to catabolic and defence processes during plant development

Within this study it was revealed that the overall functional activity of tomato leaves changes along developmental stages from anabolic processes to catabolic and defence processes, which is specifically underlines by the increase of protein degradation at later developmental stages (Table S1, 3.1.2, Table 1, Fig. 3, Table 2, 3.1.3). The high rates of anabolic processes in vegetative tissues were already shown in Arabidopsis. During cell division, high metabolic rates to sustain the synthesis of membranes, nucleic acids, and proteins were observed (Kwok & Wong, 2005; Menges et al., 2002; Smith & Atkins, 2002). In the subsequent cell expansion, chloroplast maturation and the establishment of photosynthesis were identified as the dominating processes (Andriankaja et al., 2012). The vegetative leaves of this study were defined as leaves above the first flower, thus including both proliferating and expending cells. The identified increase in catabolic and defence processes is in line with previous studies, where leaves of later developmental stages showed a decrease in photosynthesis, often accompanied by leaf senescence (Guo & Gan, 2005; Sakuraba, 2021). Leaf senescence also includes the reallocation of nutrients from old to young or reproductive tissue, thus the degradation of proteins and other macromolecules is enhanced (Buchanan-Wollaston, 1997; Guo et al., 2021; Lim et al., 2007). In this study, subtilases, insulinases, cysteine and FtsH proteases showed increased abundances along developmental stages (Fig. 3). All of them, except of insulinases, have been associated with senescence (Diaz-Mendoza et al., 2014; Guo et al., 2004; Martinez et al., 2015; Zelisko et al., 2005). Contrary to our previous study (Kleuter, Yu, Pancaldi, van der Goot, & Trindade, 2024), aspartic proteases exhibited a decrease in protein abundances, whereas transcriptomic analysis revealed an increase in gene expression for a subset of aspartic proteases along development. This discrepancy can be attributed to the detection limits of each method: transcriptomic analysis can detect low levels of mRNA, whereas the LC-MS/MS is limited to higher abundant proteins. Further, the analysis of the expression rates of genes also revealed a decrease in expression of a subset of aspartic proteases throughout development (Fig. S5). Therefore, independently from the involvement of aspartic proteases in late developmental protein degradation in Arabidopsis and Tobacco, their specific effect in tomatoes remains unclear (Diaz et al., 2008; Kato et al., 2004). The overall increasing abundance of a set of proteases observed in the here presented data aligns with a broader issue of protease activity during the protein extraction process. Proteases are undesirable, as they limit the purification yields and downstream protein functionalities through degradation, which can occur even during or after various types of leaf storage (Brouwer et al., 2023; Yu, Kleuter, de Ruijter, et al., 2023). Therefore, it can be summarized that the shift from anabolic to catabolic and defence processes within tomato leaves likely explains the decrease of protein extraction yields. Since proteases have a negative effect on protein extraction, breeding towards tomato varieties with lower overall protease activity can potentially enhance protein extraction yields.

### 4.2 Protein extractability depends on the fraction between soluble and insoluble proteins

Our previous study (Kleuter, Yu, Pancaldi, van der Goot, & Trindade, 2024) revealed a significant decline in protein extraction yield from tomato leaves, decreasing from 0.51 g/g in the vegetative stage to below 0.01 g/g [extracted protein / total protein] in the mature fruit stage. In this study, we expanded on these findings by analyzing trends of protein abundances across the four developmental stages to identify proteins contributing to the extracted fraction.

The vast majority of the proteins mass (0.730–0.760 g/g (3.2)) in tomato leaves was localized in the chloroplasts. Our data are slightly below the quantity determined by Fiorentini and Galoppini (1983), who stated that about 80 % of the total protein is located in the chloroplasts. Nevertheless, our data remains in a comparable range. Among chloroplastidic proteins, RuBisCO was the most abundant one, representing 0.141–0.202 g/g [protein/total identified protein] (Table S4). This is in line with the study of Liese et al. (2023), who reported RuBisCO to constitute 16.3 % of tomato proteins. In addition to RuBisCO, proteins associated with chlorophyll *a*-b binding, and photosystems I and II, were also particularly abundant. All these proteins participate in photosynthesis, which is the most dominant process in leaves. Interestingly, proteins involved in photosynthesis (GO:0015979) increased in concentration along tomato development (from 0.195 to 0.330 g/g [protein/identified protein]) (data not shown). This contrasts with what was observed in Arabidopsis, where photosynthesis-related proteins decreased during plant development (Tamary et al., 2019). Differences in these findings may be explained by the additional normalization to total protein per leaf area performed by Tamary et al. (2019). This normalization led to a reduction in absolute protein abundances, particularly for highly abundant proteins, which were shown to reduce in concentration along development.

In case of protein extraction from tomato leaves, it should be considered that a substantial proportion of proteins participating in photosynthesis is integrated in thylakoid membranes and their relative concentration in tomato leaves increased along development. Given that thylakoid proteins are notoriously recalcitrant to extraction (Tamayo Tenorio et al., 2018), the observed increase in these proteins likely explains the decrease in protein extraction yields as tomatoes develop.

Contrary to the insoluble fraction, a positive correlation between soluble proteins and the protein extraction yield was found (3.3). The primary component of the soluble fraction is RuBisCO, which is frequently targeted in extraction procedures due to its desirable functionalities in food and feed applications (Ducrocq et al., 2021; Pearce & Brunke, 2023; Tamayo Tenorio et al., 2016). The soluble fraction is further complemented by proteins located in the chloroplast stroma and the cytoplasm, representing easily accessible proteins which should be present in the liquid phase post leaf rupture. Thus, it can be concluded that a higher proportion of soluble proteins present in the tomato leaves enhances protein extraction yields.

### 4.3 Protein extraction yields cannot solely be explained by the abundance of proteins in the tomato leaves

Independently of the correlation between the soluble protein fraction and the protein extraction yield, the low extraction yields observed in the mature section cannot be solely attributed to a decrease in soluble proteins and an increase in insoluble proteins. Despite a soluble fraction of around 0.45–0.50 g/g [protein/total identified protein] in the leaves of later developmental stages, the extraction yield of less than 0.01 g/g [extracted protein/total protein] (Kleuter, Yu, Pancaldi, van der Goot, & Trindade, 2024) is below its potential. This suggests that protein extraction yields are likely to also depend on inhibitory molecules, such as phenolic compounds, which interfere with proteins. Phenolic compounds can form covalent bonds with proteins, particularly with the epsilon-amino group of lysine, which can lead to enhanced cross-linking of proteins. Subsequently, crosslinking can lead to changes in the protein structure that negatively affects the solubility and extractability (Anoop et al., 2023; Ozdal et al., 2013). Several phenolic compounds are involved in plant defence (Kumar et al., 2020), a process that was shown to be upregulated along developmental stages (Table 1, Table 2). Therefore, a potentially increased presence of phenolic compounds from the vegetative to the mature stage could be a reason for the lower protein extraction yield. In this perspective, an early addition of phenolic inhibitors, such as sodium metabisulfite (Liese et al., 2023) or polyvinylpolypyrrolidone (PVPP) (Laborde et al., 2006) can represent a good strategy to try to limit the negative effect of phenolic compounds on protein extraction. Nevertheless, further investigations are required to identify additional inhibitors that could improve the protein extraction yields, particularly from leaves of mature stages. In addition, reducing the presence of inhibitory molecules, such as phenolic compounds, through breeding efforts could also represent a potentially promising avenue to enhance protein extraction yields from especially older tomato leaves.

## 5 Conclusion

This study revealed that the decline in protein extraction yields from tomato leaves across developmental stages is associated with changes in protein compositions and leaf function. Proteomics analysis demonstrated a shift in protein functions from anabolic to catabolic and defence processes along the developmental stages. This transition accounts for the observed decrease in protein extraction yields through inducing the following changes in the leaves: 1) The easily extractable fraction of soluble proteins decreases along development, while the fraction of insoluble proteins increasest2) Protease activities enhance in leaves along plant development to facilitate the withdrawal of proteins from the leaves lowering the amount of protein available for extraction. 3) Inhibitory molecules accumulate over plant development, which hinder protein extraction. The next step forward to overcome the first two limitations includes centering tomato breeding towards varieties with enhanced proportions of soluble proteins and reduced expression of proteases. To resolve the third limitation, further research should prioritize identifying inhibitory molecules that lower the solubility of proteins. Once identified, these inhibitors could either be minimized through breeding or managed through optimized protein extraction protocols to minimize their impact on proteins solubility.

## Funding

This work was funded by the Wageningen University Investment theme ‘Protein Transition’.

## Declaration of generative AI and AI-assisted technologies in the writing process

During the preparation of this work the author(s) used ChatGPT from OpenAI in order to improve the manuscript's readability. After using this tool/service, the author(s) reviewed and edited the content as needed and take(s) full responsibility for the content of the publication.

## CRediT authorship contribution statement

**Marietheres Kleuter:** Writing – original draft, Visualization, Methodology, Formal analysis, Data curation, Conceptualization. **Yafei Yu:** Writing – review & editing, Conceptualization. **Lukas Verdegaal:** Writing – review & editing, Formal analysis. **Francesco Pancaldi:** Writing – review & editing, Supervision, Methodology, Formal analysis, Data curation. **Antoine H.P. America:** Writing – review & editing, Methodology. **Atze Jan van der Goot:** Writing – review & editing, Supervision. **Luisa M. Trindade:** Writing – review & editing, Supervision, Project administration, Funding acquisition, Conceptualization.

## Declaration of competing interest

The authors declare that they have no known competing financial interests or personal relationships that could have appeared to influence the work reported in this paper.

## Data Availability

Data will be made available on request.

## Appendix A Supplementary data

Supplementary material for 'Characterizing the extractable proteins from tomato leaves – a proteomics study' Image 1
