# Supplementary material for: Characterizing the extractable proteins from tomato leaves – A proteomics study
Source: Food Chem X. 2024 Dec 21;25:102114. doi: 10.1016/j.fochx.2024.102114 (PMC11741079; doi:10.1016/j.fochx.2024.102114)
Supplement: Supplementary file 1 — Supplementary material for 'Characterizing the extractable proteins from tomato leaves – a proteomics study' [file mmc1.docx]

**Supplementary data**

**
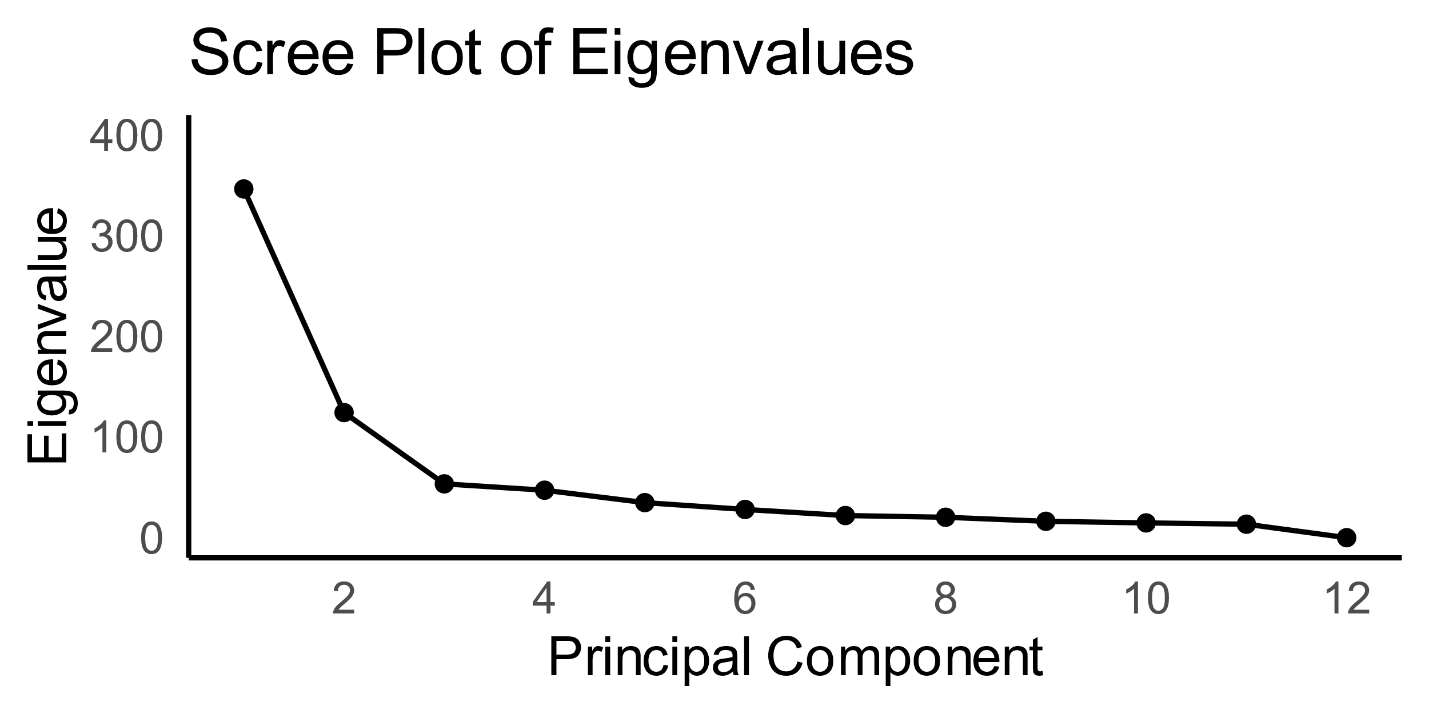
**

Figure S1: Scree plot of the Eigenvalues from every principal component within the PCA analysis.

Table S1: The 20 loadings (= proteins) having the highest and lowest values on PC 1 including their name, their location within the plant cells and the processes in which they are involved. Anabolic processes are marked in green, while catabolic processes are marked in blue.

| **Protein** | **PCA1** | **Name** | **Location** | **Involved process** |
| --- | --- | --- | --- | --- |
| Solyc07g041900.3 | 0.053 | Cysteine proteinase | Extracellular space | Protein degradation |
| Solyc06g065490.3 | 0.053 | Photosystem II reaction center PsbP family protein | Chloroplast | Photosynthesis |
| Solyc06g071920.4 | 0.053 | Glyceraldehyde-3-phosphate dehydrogenase | Cytosol | Glycolysis |
| Solyc02g064940.1 | 0.053 | Leucine-rich repeat family protein |  | Plant defence |
| Solyc11g051160.2 | 0.053 | Patellin-1 |  | Cell division, Lipid binding |
| Solyc01g087820.2 | 0.053 | Subtilisin-like protease |  | Protein degradation |
| Solyc03g119860.3 | 0.053 | Peptidyl-prolyl cis-trans isomerase |  | Protein folding |
| Solyc03g114150.3 | 0.053 | Aldehyde dehydrogenase |  | NAD+ activity |
| Solyc12g094620.3 | 0.053 | Catalase | Cytosol, plasma membrane, peroxisome | Stress response |
| Solyc08g079870.3 | 0.053 | Subtilisin |  | Protein degradation |
| Solyc12g006140.2 | 0.053 | Cab-5 gene encoding chlorophyll a/b-binding protein | Chloroplast | Photosynthesis |
| Solyc01g006300.3 | 0.053 | LECEVI1A, peroxidase | Extracellular space | Stress response |
| Solyc01g102660.4 | 0.053 | Glutathione S-transferase | Cytoplasm |  |
| Solyc08g080640.2 | 0.053 | NP24 protein precursor |  |  |
| Solyc10g052880.1 | 0.053 | Inactive leucine-rich repeat receptor-like protein kinase | Plasma membrane | Plant defence |
| Solyc11g066590.2 | 0.053 | Serine carboxypeptidase S28 family protein |  | Protein degradation |
| Solyc02g081170.4 | 0.052 | Lipid associated protein CHRC | Plastid |  |
| Solyc06g048410.4 | 0.052 | Iron superoxide dismutase | Chloroplast nucleoid | Stress response |
| Solyc03g082890.3 | 0.052 | Thylakoid lumenal 17.4 kDa protein | Chloroplast |  |
| Solyc01g008620.4 | 0.052 | Glucan endo-1,3-beta-glucosidase | Plasma membrane | Plant defence |
|  | | | | |
| Solyc09g008280.2 | -0.051 | S-adenosyl-L-methionine synthetase Z24743 | Cytosol | Amino acid synthesis |
| Solyc10g006900.3 | -0.051 | Light dependent NADH protochlorophyllide oxidoreductase 3 s2 | Chloroplast | Photosynthesis |
| Solyc11g066410.2 | -0.051 | 50S ribosomal protein L9 | Chloroplast | Protein synthesis |
| Solyc02g077990.3 | -0.051 | 30S ribosomal protein S5 | Chloroplast | Protein synthesis |
| Solyc08g083350.4 | -0.051 | 50S ribosomal protein L11 | Chloroplast | Protein synthesis |
| Solyc07g006000.3 | -0.051 | 50S ribosomal protein L35 | Chloroplast | Protein synthesis |
| Solyc04g009200.3 | -0.051 | Glutamate 1-semialdehyde 2,1-aminomutase | Chloroplast | Chlorophyll biosynthesis |
| Solyc01g103450.3 | -0.052 | 70 kDa heat shock protein | Cytoplasm, chloroplast | Purine metabolism |
| Solyc00g500047.1 | -0.052 | 50S ribosomal protein L2 | Chloroplast | Protein synthesis |
| Solyc04g009540.1 | -0.052 | 50S ribosomal protein L10 | Chloroplast | Protein synthesis |
| Solyc03g115980.1 | -0.052 | Geranylgeranyl diphosphate reductase | Chloroplast |  |
| Solyc10g081510.2 | -0.052 | Ethylene-responsive methionine synthase |  | Amino acid synthesis |
| Solyc12g042060.3 | -0.052 | ATP-dependent Clp protease ATP-binding subunit ClpC | Chloroplast | ATP binding, protein degradation |
| Solyc10g008740.3 | -0.052 | Mg-protoporphyrin IX chelatase | Chloroplast | Photosynthesis |
| Solyc01g028810.3 | -0.052 | Beta chaperonin 60 | Chloroplast | RuBisCO |
| Solyc03g120850.4 | -0.053 | Chaperonin 60 beta | Chloroplast | RuBisCo |
| Solyc11g068820.3 | -0.053 | 50S ribosomal protein L27 | Chloroplast | Protein synthesis |
| Solyc11g069790.2 | -0.053 | 60 kDa chaperonin | Chloroplast | RuBisCO |
| Solyc04g054740.3 | -0.053 | Myo-inositol-1-phosphate synthase | Cytoplasm | Myo-inositol biosynthesis |
| Solyc01g057830.3 | -0.053 | 30S ribosomal protein S1 protein | Chloroplast | Protein synthesis |


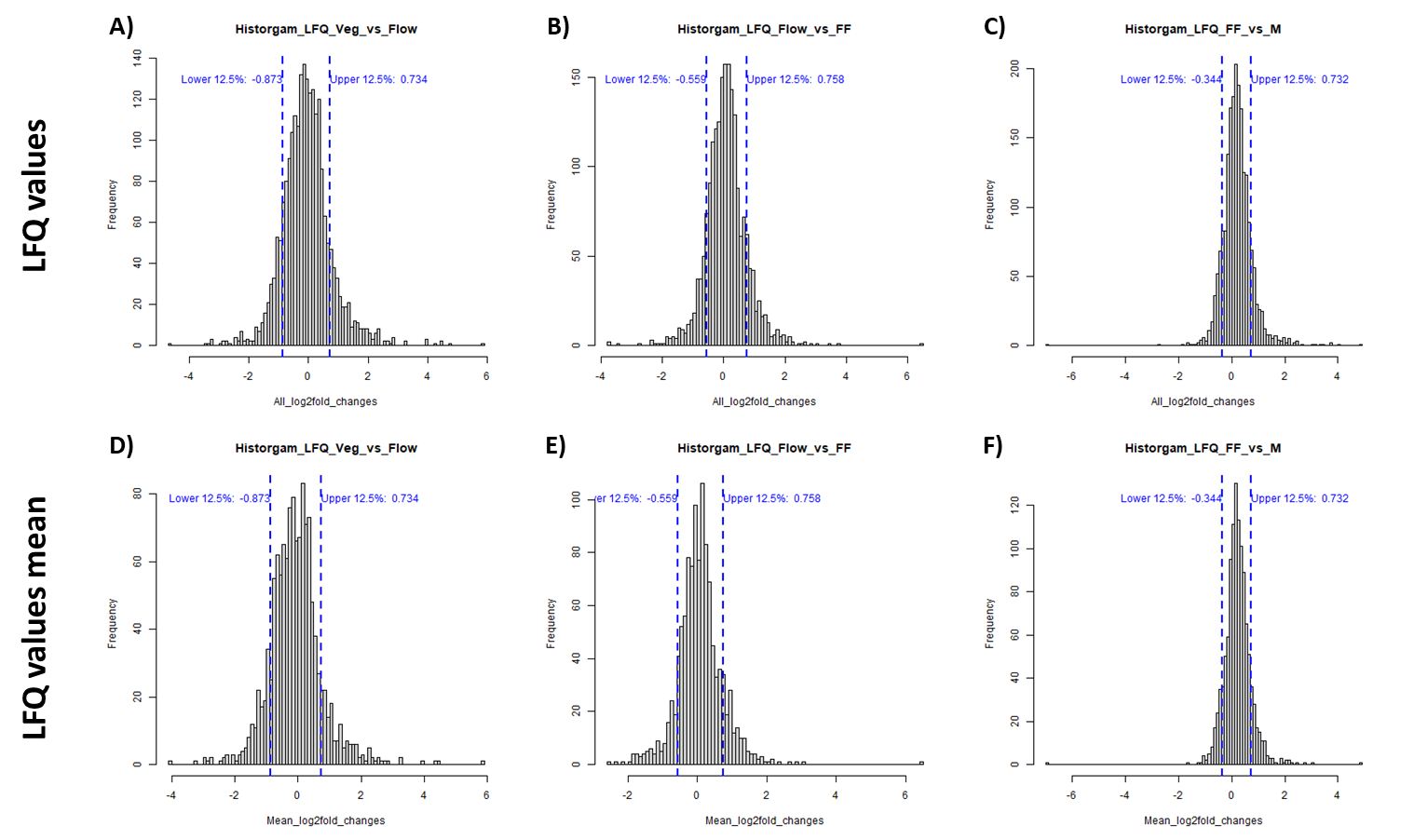


Figure S2: Distribution of the log2 fold changes per transitional stage (Vegetative to Flowering – A, D; Flowering to Fruit-forming – B, E; Fruit-forming to mature – C, F) of separated biological replicates (A, B, C) and for their means (D, E, F).

Table S2: 53 up- and 26 downregulated genes received from the Venn diagram and further used for the enrichment analysis.

| **Upregulated genes** | **Downregulated genes** |
| --- | --- |
| Solyc01g006300.3 | Solyc12g096700.2 |
| Solyc09g090980.3 | Solyc03g120850.4 |
| Solyc04g071900.4 | Solyc10g008740.3 |
| Solyc01g106620.2 | Solyc04g054740.3 |
| Solyc10g055800.2 | Solyc03g121070.3 |
| Solyc12g099650.2 | Solyc01g028810.3 |
| Solyc01g105410.4 | Solyc06g072490.3 |
| Solyc10g075070.3 | Solyc01g099410.3 |
| Solyc01g087820.2 | Solyc03g115980.1 |
| Solyc01g087800.2 | Solyc10g086150.2 |
| Solyc07g062490.1 | Solyc08g076970.3 |
| Solyc03g082720.3 | Solyc00g500047.1 |
| Solyc08g079870.3 | Solyc07g064950.3 |
| Solyc08g079900.3 | Solyc06g008260.3 |
| Solyc01g059965.1 | Solyc11g006250.2 |
| Solyc06g071920.4 | Solyc01g067740.3 |
| Solyc08g013670.3 | Solyc01g079090.4 |
| Solyc09g007010.1 | Solyc08g069030.4 |
| Solyc03g111010.4 | Solyc10g075100.2 |
| Solyc10g055810.2 | Solyc01g008960.3 |
| Solyc01g008620.4 | Solyc08g007140.4 |
| Solyc02g064940.1 | Solyc08g077880.3 |
| Solyc08g080640.2 | Solyc02g067080.4 |
| Solyc11g051160.2 | Solyc10g005110.3 |
| Solyc01g006290.4 | Solyc10g081440.2 |
| Solyc02g081170.4 | Solyc01g009420.4 |
| Solyc02g082920.4 |  |
| Solyc11g069430.2 |  |
| Solyc01g006555.1 |  |
| Solyc11g066590.2 |  |
| Solyc10g052880.1 |  |
| Solyc02g077040.4 |  |
| Solyc07g009380.4 |  |
| Solyc07g005100.4 |  |
| Solyc01g102660.4 |  |
| Solyc01g097240.3 |  |
| Solyc03g083910.5 |  |
| Solyc04g054980.3 |  |
| Solyc01g094790.3 |  |
| Solyc07g041900.3 |  |
| Solyc10g083290.4 |  |
| Solyc05g050130.4 |  |
| Solyc07g022900.4 |  |
| Solyc07g062700.3 |  |
| Solyc10g055200.1 |  |
| Solyc07g053970.3 |  |
| Solyc02g083410.4 |  |
| Solyc02g068500.3 |  |
| Solyc01g112000.4 |  |
| Solyc02g082930.3 |  |
| Solyc02g077880.3 |  |
| Solyc04g079440.3 |  |
| Solyc02g076980.5.1 |  |

Table S3: List of the 30 most abundant proteins as well as their functional category and location. The abundance of the proteins is listed throughout all developmental stages (Veg = vegetative, Flow = flowering, FF = fruit-forming, M= Mature fruit)

| Protein | Functional category | Location | Veg | Flow | FF | M |
| --- | --- | --- | --- | --- | --- | --- |
| Solyc00g500024.1 | Photosystem_II | Chloroplast | 0.0113 | 0.0116 | 0.0155 | 0.0139 |
| Solyc00g500050.1 | ATP_synthase | Chloroplast | 0.0181 | 0.0139 | 0.0103 | NA |
| Solyc00g500054.1 | Photosystem_II | Chloroplast | 0.0195 | 0.0234 | 0.0264 | 0.0248 |
| Solyc00g500056.1 | DNA_ polymerase |  | 0.0125 | 0.0111 | 0.0122 | 0.0145 |
| Solyc00g500063.1 | RuBisCO | Chloroplast | 0.1229 | 0.1386 | 0.0893 | 0.0927 |
| Solyc00g500064.1 | unknown_protein | Chloroplast | 0.0146 | 0.0144 | NA | NA |
| Solyc00g500130.1 | Photosystem_II | Chloroplast | 0.0143 | 0.0157 | 0.0169 | 0.0155 |
| Solyc01g105050.3 | Chlorophyll_a-b_binding | Chloroplast | 0.0087 | 0.0116 | 0.0156 | 0.0127 |
| Solyc02g065400.3 | Photosystem_II | Chloroplast | 0.0105 | 0.0139 | 0.0149 | 0.0134 |
| Solyc02g069460.3 | Photosystem_I | Chloroplast | 0.0093 | 0.0103 | 0.0107 | 0.0117 |
| Solyc02g071000.1 | Chlorophyll_a-b_binding | Chloroplast | 0.0478 | 0.0564 | 0.0682 | 0.0704 |
| Solyc02g079950.3 | Photosystem_II | Chloroplast | 0.0195 | 0.0184 | 0.0217 | 0.0189 |
| Solyc03g005780.3 | Chlorophyll_a-b_binding | Chloroplast | 0.0121 | 0.0158 | 0.0218 | 0.0164 |
| Solyc03g034220.3 | RuBisCO | Chloroplast | 0.0286 | 0.0634 | 0.0517 | 0.0836 |
| Solyc04g009030.3 | Glycolysis | Cytoplasm | 0.0214 | 0.0240 | 0.0185 | 0.0148 |
| Solyc04g082010.1 | Photosystem_I | Chloroplast | 0.0120 | 0.0119 | 0.0117 | 0.0116 |
| Solyc05g052520.3 | Protein_ phosphatase |  | 0.0183 | 0.0142 | 0.0134 | 0.0128 |
| Solyc06g005060.3 | EF1alpha | Cytoplasm | 0.0102 | NA | NA | NA |
| Solyc06g054260.1 | Photosystem_I | Chloroplast | 0.0094 | NA | 0.0097 | NA |
| Solyc06g063370.3 | Chlorophyll_a-b_binding | Chloroplast | 0.0197 | NA | 0.0261 | 0.0243 |
| Solyc06g082940.3 | Photosystem_I | Chloroplast | 0.0101 | NA | 0.0122 | 0.0114 |
| Solyc07g041720.1 | Programmed_cell_ death | Extracellular space | 0.0170 | NA | 0.0268 | 0.0224 |
| Solyc07g044860.3 | Photosystem_II | Chloroplast | 0.0159 | NA | 0.0203 | 0.0185 |
| Solyc07g066310.3 | Photosystem_II | Chloroplast | 0.0087 | 0.0087 | 0.0116 | NA |
| Solyc09g010400.3 | Histone | Nucleus | 0.0097 | NA | NA | NA |
| Solyc09g014520.3 | Chlorophyll_a-b_binding | Chloroplast | 0.0126 | 0.0140 | 0.0165 | 0.0164 |
| Solyc09g063130.3 | Photosystem_I | Chloroplast | 0.0083 | NA | NA | NA |
| Solyc10g086580.2 | RuBisCO_activase | Chloroplast | 0.0238 | 0.0221 | 0.0148 | 0.0087 |
| Solyc11g067110.2 | DNA_polymerase |  | 0.0423 | 0.0547 | 0.0628 | 0.0381 |
| Solyc12g006140.2 | Chlorophyll_a-b_binding | Chloroplast | 0.0091 | 0.0195 | 0.0289 | 0.0312 |
| Solyc01g081510.4 | NAD/NADP_ reductase |  | NA | 0.0082 | 0.0131 | 0.0108 |

| Solyc06g054260.1 | EF1alpha | Cytoplasm | NA | 0.0081 | NA | NA |
| --- | --- | --- | --- | --- | --- | --- |
| Solyc06g063370.3 | Photosystem_I | Chloroplast | NA | 0.0229 | NA | NA |
| Solyc06g082940.3 | Chlorophyll_a-b_binding | Chloroplast | NA | 0.0101 | NA | NA |
| Solyc07g041720.1 | Photosystem_I | Chloroplast | NA | 0.0167 | NA | NA |
| Solyc07g044860.3 | Programmed_cell_ death | Extracellular_ space | NA | 0.0165 | NA | NA |
| Solyc08g013670.3 | Photosystem_I | Chloroplast | NA | 0.0104 | 0.0190 | 0.0200 |
| Solyc10g007690.3 | Chlorophyll_a-b_binding | Chloroplast | NA | 0.0080 | NA | NA |
| Solyc08g080640.2 | Defence_protein | Cytoplasm/ Vacuole | NA | NA | 0.0101 | 0.0119 |
| Solyc09g007010.1 | Defence_protein | Cytoplasm/ Vacuole | NA | NA | 0.0212 | 0.0197 |
| Solyc03g115900.4 | Chlorophyll_a-b_binding | Chloroplast | NA | NA | NA | 0.0088 |
| Solyc10g075150.2 | Lipid_transport |  | NA | NA | NA | 0.0118 |
| Solyc12g099650.2 | Photosystem_II | Chloroplast | NA | NA | NA | 0.0097 |

Table S4: Abundance of proteins which were clustered by functional category in g/g [protein/total identified protein] throughout the four developmental stages vegetative, flowering, fruit-forming and mature.

| **Functional category** | **Vegetative** | **Flowering** | **Fruit-forming** | **Mature** |
| --- | --- | --- | --- | --- |
| Histone | 0.0097 | NA | NA | NA |
| Lipid_transport | NA | NA | NA | 0.0118 |
| EF1alpha | 0.0102 | 0.0081 | NA | NA |
| unknown_protein | 0.0146 | 0.0144 | NA | NA |
| NAD/NADP_reductase | NA | 0.0082 | 0.0131 | 0.0108 |
| ATP_synthase | 0.0181 | 0.0139 | 0.0103 | NA |
| Protein_phosphatase | 0.0183 | 0.0142 | 0.0134 | 0.0128 |
| Defence_protein | NA | NA | 0.0313 | 0.0316 |
| RuBisCO_activase | 0.0238 | 0.0221 | 0.0148 | 0.0087 |
| Glycolysis | 0.0214 | 0.0240 | 0.0185 | 0.0148 |
| Programmed_cell_death | 0.0170 | 0.0165 | 0.0268 | 0.0224 |
| Photosystem_I | 0.0491 | 0.0721 | 0.0632 | 0.0546 |
| DNA_polymerase | 0.0548 | 0.0658 | 0.0750 | 0.0525 |
| Photosystem_II | 0.0998 | 0.0918 | 0.1272 | 0.1147 |
| Chlorophyll_a-b_binding | 0.1101 | 0.1354 | 0.1771 | 0.1802 |
| RuBisCO | 0.1515 | 0.2021 | 0.1409 | 0.1764 |

Table S5: Number of proteins belonging to each of the 16 functional categories used in the abundance quantification of the 30 most abundant proteins.

| **Functional category** | **Vegetative** | **Flowering** | **Fruit-forming** | **Mature** |
| --- | --- | --- | --- | --- |
| Histone | 1 | *NA* | *NA* | *NA* |
| Lipid_transport | *NA* | *NA* | *NA* | 1 |
| EF1alpha | 1 | 1 | *NA* | *NA* |
| unknown_protein | 1 | 1 | *NA* | *NA* |
| NAD/NADP_reductase | *NA* | 1 | 1 | 1 |
| ATP_synthase | 1 | 1 | 1 | *NA* |
| Protein_phosphatase | 1 | 1 | 1 | 1 |
| Defence_protein | *NA* | *NA* | 2 | 2 |
| RuBisCO_activase | 1 | 1 | 1 | 1 |
| Glycolysis | 1 | 1 | 1 | 1 |
| Programmed_cell_death | 1 | 1 | 1 | 1 |
| Photosystem_I | 5 | 5 | 5 | 4 |
| DNA_polymerase | 2 | 2 | 2 | 2 |
| Photosystem_II | 7 | 6 | 7 | 7 |
| Chlorophyll_a-b_binding | 6 | 7 | 6 | 7 |
| RuBisCO | 2 | 2 | 2 | 2 |


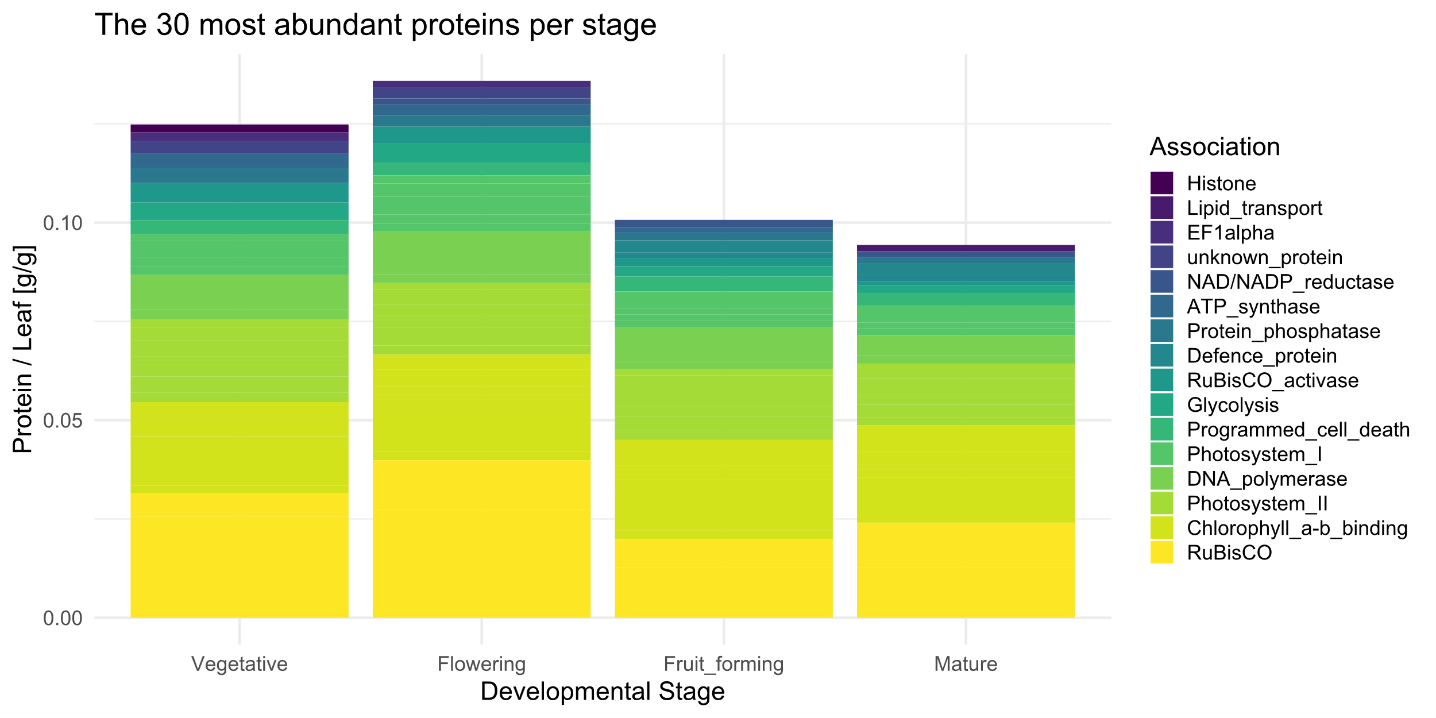


Figure S3: The abundance of the top 30 proteins shown as protein/dry leaf [g/g] per developmental stage (Vegetative, Flowering, Fruit-forming, Mature). Proteins were associated to 16 different groups, based on their activity (Table S5).


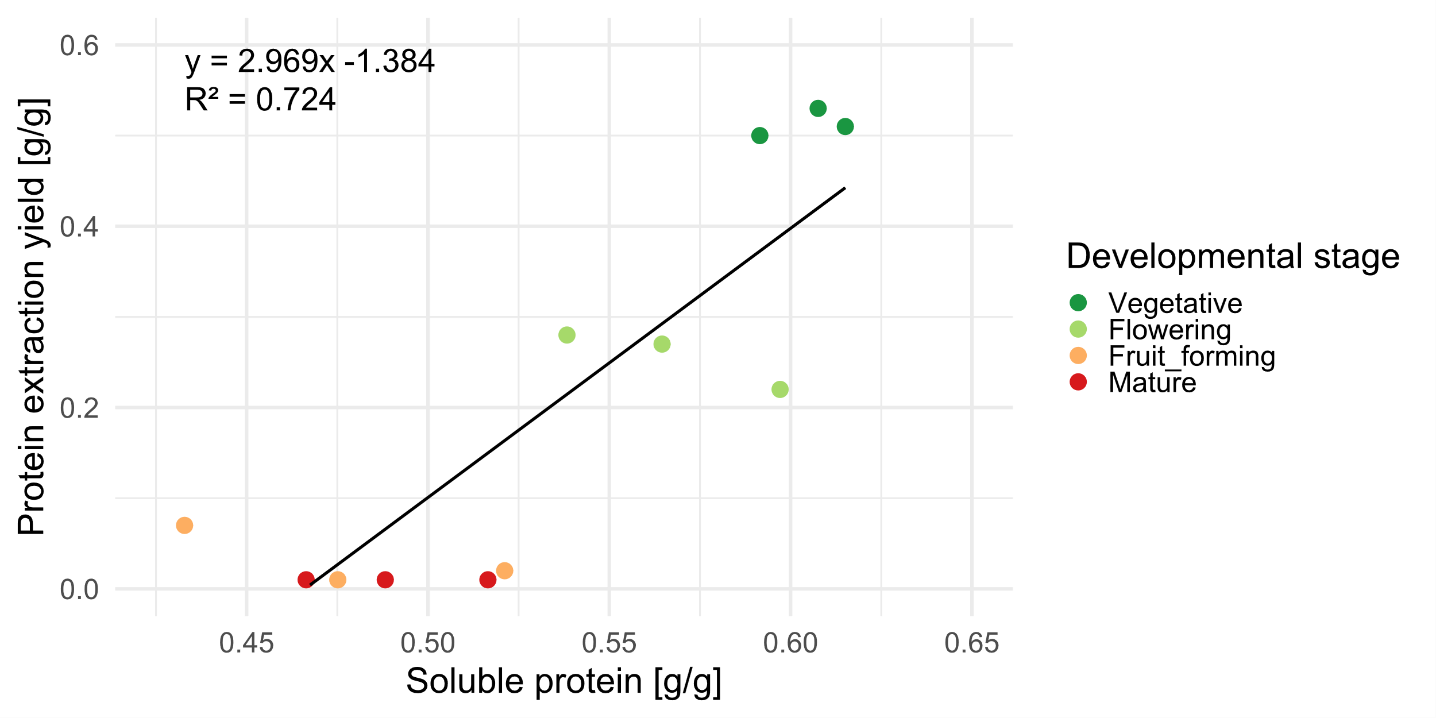


Figure S4: A correlation analysis between the protein extraction yield [g/g] and the soluble proteins [g/g]. The protein extraction yields were taken from (Kleuter et al. 2024b). The soluble protein was defined as proteins annotated by the cellular component GO terms cytoplasm or chloroplast stroma. The plot shows three biological replicates across the four developmental stages (dark green = Vegetative, light green = Flowering, orange = Fruit-forming, red = Mature). The linear regression and its correlation are also displayed.


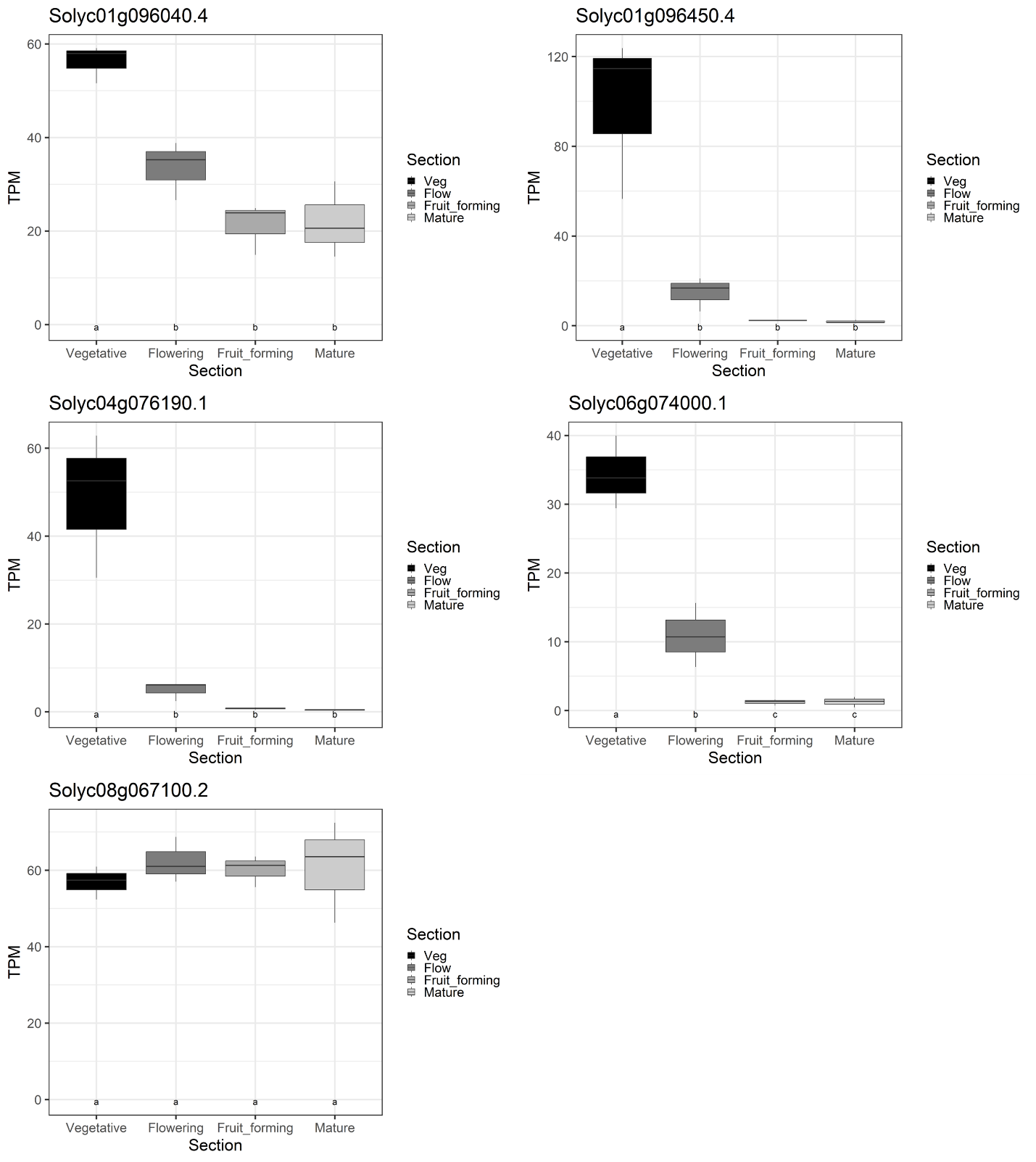


Figure S5: Expression values of aspartic proteases, shown as transcripts per million (TPM) across the four developmental stages (Vegetative, Flowering, Fruit_forming, Mature). Letters indicate the statistically significant differences, defined by a threshold of p < 0.05.
